# Development of a health literacy scale for preconception care: a study of the reproductive age population in Japan

**DOI:** 10.1186/s12889-021-12081-0

**Published:** 2021-11-10

**Authors:** Maiko Suto, Haruhiko Mitsunaga, Yuka Honda, Eri Maeda, Erika Ota, Naoko Arata

**Affiliations:** 1 Department of Health Policy, National Center for Child Health and Development, 10-1 Okura 2-chome, Setagaya, Tokyo, 157-8535 Japan; 2 Graduate School of Education and Human Development, Nagoya University, Furo-cho, Chikusa-ku Nagoya, 464-8601 Japan; 3 Division of Maternal Medicine, Center for Maternal-Fetal, Neonatal and Reproductive Medicine, National Center for Child Health and Development, 10-1 Okura 2-chome, Setagaya, Tokyo, 157-8535 Japan; 4 Department of Environmental Health Sciences, Akita University Graduate School of Medicine, 1-1-1 Hondo, Akita, 010-8543 Japan; 5 Global Health Nursing, Graduate School of Nursing Science, St. Luke’s International University, 3-6-2 Tsukiji, Chuo-ku, Tokyo, 104-0045 Japan

**Keywords:** Preconception care, Health literacy, Scale development, Population-based approach, Japanese

## Abstract

**Background:**

Preconception care aims to improve both maternal and child health in the short as well as long term, along with providing health benefits to adolescents, women, and men, whether or not they plan to become parents. However, there is limited evidence regarding the effectiveness of interventions for improving preconception health in population-based settings. To accumulate evidence in this field, this study focused on the concept of health literacy, and aimed to develop a self-report health literacy scale in Japanese, focusing on preconception care.

**Methods:**

We conducted a cross-sectional online survey. Participants were recruited from December 2019 to February 2020 from the registered members of a web-based research company. Participants were Japanese men and women aged 16–49 (*n* = 2000). A factor analysis was conducted to select both factors and items for health-related behavior and skills (33 initial items were generated), along with an item response theory analysis to examine how the 16 items were related to people’s knowledge of preconception care.

**Results:**

We developed a 6-factor (including “appropriate medical examinations,” “appropriate diet,” “stress coping,” “healthy weight,” “safe living environment,” and “vaccinations”), 25-item behavior and skills scale, as well as a 13-item knowledge scale, to evaluate participants’ health literacy around preconception care. A shortened version, consisting of 17 items, was also prepared from the 25 items. The reliability coefficients of total scores and each factor of the behavior and skills scale were comparatively high, with weak-to-moderate correlation between behavior and skills and knowledge.

**Conclusions:**

The new scale will, ideally, provide information on the current state of preconception care health literacy of the general population. In addition, this scale, which consists of both behavioral/skills and knowledge dimensions, should help support the effective implementation of risk assessment programs and interventions aimed at promoting behavioral changes using a population-based approach. Future studies using different question/administration formats for diverse populations, and considering respondents’ opinions on health literacy scales should be effective in improving this scale.

**Supplementary Information:**

The online version contains supplementary material available at 10.1186/s12889-021-12081-0.

## Background

Preconception care aims to improve both maternal and child health in the short and long term by improving the health statuses of women and couples before pregnancy, while reducing behavioral and environmental factors that could become future maternal and child health risks [1]. Lifestyle habits, such as drinking alcohol, smoking, drug use, high-risk sexual behavior, and the nutrition (e.g., obesity and lack of essential vitamins) of parents before/during pregnancy, have been found to affect the health of future mothers and children. They are known to trigger gestational diabetes and hypertensive disorders during pregnancy, and low birthweight, preterm birth, birth defects, obesity and chronic disease in offspring, making it necessary to make women and men of reproductive age aware of these behaviors and of their influence on reproductive health and childbearing [2–4]. The greatest impact occurs in early pregnancy, often before women know that they are pregnant, and considering the substantial time needed to reach a healthy lifestyle, intervention before pregnancy is recommended [2, 4]. In this study, we defined preconception care as interventions provided to women and couples of childbearing age to improve maternal and child health in the short and long term; simultaneously, preconception care improving their health statuses provides health benefits to both adolescent and adult women and men, whether or not they plan to be parents [5]. The World Health Organization (WHO) and the Centers for Disease Control and Prevention (CDC) advocate the importance of pre-pregnancy healthcare and the need for a provision of comprehensive information and professional health services [1, 6].

However, there is limited evidence regarding effective interventions for preconception health in the primary care setting or in the public health and community settings [4, 7, 8]. To accumulate knowledge in this field, more evidence is needed regarding the effectiveness of interventions aimed at the general lifestyles and behavioral risks for the general population in terms of preconception care [9]. To achieve this, the present study focused on the concept of health literacy. Although, there is no consensus about the definition of health literacy or its conceptual dimensions [10], in this study, we defined health literacy as the knowledge and competency to access, understand, appraise, and apply health-related information; the competencies also incorporate the qualities of functional, interactive and critical health literacy as proposed by Nutbeam [10, 11]. Health literacy is considered an asset for improving people’s empowerment in the areas of healthcare, disease prevention, and health promotion, and through the health literacy process, people are expected to be able to take control of their own health by applying their specific health literacy skills [10]. It is important to enhance health literacy, specifically within the preconception care setting wherein risk assessment and reduction programs are implemented to reduce lifestyle-related problems, such as alcohol consumption, smoking, and under- or over- nutrition before pregnancy, in addition to promoting future maternal and child health.

To improve people’s health literacy, it is necessary to properly evaluate their competencies and the effectiveness of intervention programs. Evaluations for preconception care have been conducted in terms of health knowledge(e.g., folate can prevent birth defects), behavioral changes (such as folate intake, alcohol, and smoking), and health outcomes (e.g., adverse pregnancy outcomes, such as low birth weight and preterm birth) [7, 8]. However, there are no appropriate indicators to measure health literacy specific to preconception care among the general population. There are scales, such as comprehensive concept-based health literacy scale for the general population, [12] also in Japanese [13], as well as health literacy measurement tools that focus on specific aspects, such as the recognition and pronunciation of medical terms, numeracy, comprehension, and decision-making competencies [12]. There are scales that cover a comprehensive range of skills including functional, communicative/interactive, and critical health literacy in the clinical and public health contexts [14–16]. However, these are designed for general use or are specific to diabetic patients and have limited capacity to assess the specific skills in preconception care. Kawata et al. developed a health literacy scale for women of reproductive age comprising the following factors: women’s choice for adopting health information and practice, self-care during menstruation, knowledge of the female body, and sexual discussions with partner [17]. However, no study has developed a health literacy scale focused on preconception care for men and women of reproductive age. The health literacy scale specific to preconception care is needed for assessment and reduction of risks related to short- and long-term maternal and child health outcomes, a key issue in preconception care. Therefore, this study aimed to develop a self-report health literacy scale in Japanese, to assess the knowledge and skills necessary to acquire information, focusing specifically on preconception care, understanding this information, and critically analyzing and appraising it. The use of this scale should allow measuring the health literacy of both women and men of reproductive age, along with the evaluation of interventions occurring within the primary care setting or public health and community setting.

## Methods

We defined health literacy as knowledge and competencies to access, understand, appraise, and apply health-related information. We considered health knowledge as a domain of health literacy, and developed the scale as divided into items on knowledge and behavior/skills related to the health literacy competencies. We conducted a factor analysis to select the factors and items for a health literacy scale relating to people’s behavior and skills. In addition, to develop more readily available screening tools, a shortened version with a reduced number of items was also examined. Items relating to people’s knowledge of preconception care were analyzed using the item response theory (IRT) to ensure that the scale contained items with varying difficulties, and that items had sufficient capacity to differentiate between participants. This study follows the COSMIN reporting guideline [18].

### Participants

We conducted a cross-sectional online survey. Participants were recruited from December 2019 to February 2020 from the registered members of a web-based research company (commissioned by Cross Marketing Group, Inc.). In this study, we targeted a wide range of age groups, both men and women, with the aim of developing the proposed measurement tool in universal environments, such as in schools and workplaces. Inclusion criterion was men and women aged 16–49 years. The survey included participants across Japan, with the extracted sample adjusted for their sex, place of residence, and age. We designed it so that 30% of all participants were pregnant or had delivered a child at some point in the past (aimed to include people with a broad knowledge level about pregnancy and childbirth).

Participants were involved via e-mail with a link to the response Webpage for survey cooperation. They were asked to respond only if they agreed to participate in this study. The survey was first administered to 1000 participants, then to a different set of 1000 participants, to examine if the original findings could be replicated with different samples (resulting in a total of 2000 participants).

### Questionnaire design

#### The health literacy scale items

Contents of behavior/skills and knowledge for the preconception care scale were extracted from the relevant literature to create a first items’ pool, with specialists in maternal medicine developing each questionnaire item. The following topics were identified: reproductive life plans, nutrition, physical activity, folic acid supplementation, reproductive health, sexually transmitted diseases, immunizations, infectious diseases, environmental exposures, psychosocial stressors, mental health, tobacco use, alcohol and other substance use, partner violence, chronic medical conditions, medication use, family and genetic history, and regular checkups [5, 19, 20]. The questionnaire was designed to cover four domains of health literacy competencies (access, understanding, appraisal, and application of health information). Questions were phrased as: “Do you read …? ” (examples of Access competency); “Do you know …? ” (examples of Understanding competency); “Are you able to assess …? ” (examples of Appraisal competency); and “Do you make it a habit …? ” (examples of Application competency). Questions on respondents’ behavior and skills were answerable via a 4-point Likert scale (1 = “strongly true,” 2 = “somewhat true,” 3 = “rarely true,” and 4 = “not at all”). For the questions on respondents’ knowledge around preconception care, the participants were asked to choose one correct answer from a range of multiple options.

#### Socioeconomic variables

Participants’ current place of residence, sex, age, marital status, parity (including if currently pregnant), employment, education history, and annual household income were surveyed.

#### Health status

Data were collected on respondents’ height, weight, sleep duration, current health status, regular medical screenings, plans to become pregnant within a year, alcohol use, and whether or not the participant smoked.

The Communicative and Critical Health Literacy (CCHL) scale is a 5-item, 5-point (theoretical range: 1–5) health literacy scale, with higher scores indicating higher health literacy. It has a Cronbach’s α of 0.86 according to a previous study [14]. Additionally, the Japanese version of the Cardiff Fertility Knowledge Scale, a 13-item scale for assessing fertility knowledge was used. Correct answers are assigned one point and incorrect or “do not know” answers are assigned zero points; scores are then reported as a percentage of the highest possible score. This scale has a Cronbach’s α of 0.74 based on a previous study [21]. Both of these scales were used to verify the validity of our questionnaire. We adopted these scales for criterion-related validity verification because they are both validated in Japanese and contain a relatively small number of items.

### Data analysis

#### Factor analysis for the health literacy behavior and skills of preconception care

An exploratory factor analysis was conducted to select the factors and items for the health literacy scale relating to people’s behavior and skills. We used 33 items that sought respondents’ answers on their behavior and skills using a 4-point Likert scale. Cronbach’s alpha coefficients were calculated for the factors of each scale to determine their reliability. In addition, in consideration of practicality, a shortened version, with a reduced number of items, was also examined. We permitted correlation among factor score scales, so we used promax rotation as a method of oblique rotation.

We used a two-way analysis of variance (ANOVA) to examine sex, history of pregnancy, and childbirth related differences in the subscale obtained from the factor analysis. There were a few participants (or their partners) who were currently pregnant, so we excluded them from this ANOVA.

#### IRT analysis for the knowledge scale

In total, 13 questions relating to respondents’ knowledge of preconception care (that asked them to choose one answer from a range of options) were summarized as dichotomous data, which marked each response to these items as either correct (1) or incorrect (0). Next, the dichotomous data were checked to see if the scale was unidimensional using factor analysis, with the results then demonstrating the unidimensionality of the scale. Then, the dichotomous data were analyzed using IRT to obtain the item difficulty index, which excluded the distribution effect of participants’ knowledge levels; the scale needed to include items with varying difficulties and to have sufficient capacity to differentiate between participants. We assumed a two-parameter logistic model (2PL) to examine the discrimination parameters (slope: how well the items differentiated between participants with low and high level of knowledge of preconception care) and difficulty parameters (threshold: the item’s difficulty with larger threshold estimate indicated greater difficulty) of each item. Then, we compared these with those of the Cardiff Fertility Knowledge Scale. Additionally, expecting a significant difference in the correct response rates for each item between men and women, we analyzed the data using a multiple group IRT model, with women as the reference group.

#### Confirmation of scale reliability and validity

Cronbach’s α was calculated to assess internal consistency of scores for each scale. To confirm the validity of the scale, we conducted a correlation analysis using the variables of the behavior and skills and the knowledge scales of preconception care. Pearson’s r was calculated between the subscale and total scores obtained from the factor analysis using items for respondents’ behavior and skills; the scores of participants’ knowledge of preconception care were scaled by the IRT analysis, the related scales of the CCHL scale scores, and Cardiff Fertility Knowledge Scale scores to confirm the criterion validity (Table 5). As significant differences in the knowledge scores between men and women were predicted, we conducted the analysis by sex.

The significance level was set at *p* < 0.05. The data were analyzed using statistical packages SPSS 26.0 (factor analysis), BILOG-MG 3 (IRT), and AMOS 26.0 (multivariate regression with a multiple population simultaneous analysis).

## Results

### Participant characteristics

The characteristics (*n* = 2000) are described in Table 1 (Additional file 1 shows the first and second group results, separately). There were 1000 men and women each, of average age of 34.8 years (standard deviation (SD) ±9.1). Although no sex differences were found in most of the items, men were more likely than women to work full-time, have an educational level of “universities and graduate schools,” and drink or smoke. Fewer women had regular checkups “every year” than men (63.9% men and 49.9% women).

**Table 1:** Characteristics of study participants

	(*n* = 2000)	Men		Women	
Socioeconomic attributes		n	%	n	%
Age (years)	16–19	49	4.9	42	4.2
	20–29	283	28.3	290	29.0
	30–39	334	33.4	334	33.4
	40–49	334	33.4	334	33.4
Marital status	Yes	343	34.3	403	40.3
	No	657	65.7	597	59.7
Parity	Yes	278	27.8	271	27.1
	No	708	70.8	700	70.0
	Currently pregnant	14	1.4	29	2.9
Employment	Full Time	710	71.0	429	42.9
	Part Time	84	8.4	208	20.8
	No work/In School	113	11.3	78	7.8
	No work/No study	93	9.3	285	28.5
Final education	Junior/High schools	261	26.1	335	33.5
	Vocational/Junior colleges	201	20.1	343	34.3
	Universities/Graduate schools	538	53.8	322	32.2
Annual household income (Yen)	Less than 2 million	128	12.8	189	18.9
	2 Less than - 5 million	370	37.0	430	43.0
	5 Less than - 10 million	308	30.8	250	25.0
	10 million or more	81	8.1	53	5.3
	Students	113	11.3	78	7.8
**Health status**		n	%	n	%
Body Mass Index	Mean (SD)	22.8	3.9	21.1	4.2
Hours of sleep per day	Median (25–75 percentile)	6	6–7	6	6–7
Current health status	Very well	187	18.7	152	15.2
	Moderately well	274	27.4	287	28.7
	Normal	372	37.2	369	36.9
	Slightly well	129	12.9	158	15.8
	Not at all well	38	3.8	34	3.4
Regular health checkups	Yearly visit	639	63.9	499	49.9
	Occasional visit	129	12.9	182	18.2
	No visit	232	23.2	319	31.9
Current desire for pregnancy	Yes	124	12.4	156	15.6
	No	876	87.6	844	84.4
Current drinking	Yes	421	44.3	250	26.1
	No	530	55.7	708	73.9
Current smoking	Yes	285	30.0	135	14.1
	No	666	70.0	823	85.9

*"Current drinking and smoking” excludes participants under 20 years old.

### Factor analysis results for the health literacy behavior and skills of preconception care

The explanatory factor analysis was conducted using all 33 items relating to people’s behavior and skills. Each item was evaluated on a 4-point scale (maximum likelihood estimation method with a promax rotation). To achieve a simple structure, we changed the number of factors and excluded items with low commonality (< 0.1) and those that overlapped with multiple factors with a high factor loading (> 0.3, < -0.3). As a result, six factors, comprising 25 items, were retained (Table 2; excluded items are shown in Additional files 2 and 3). Then, the final 25 items were matched in the first and second surveys. We labeled the extracted factors as “appropriate medical examinations” (seven items), “appropriate diet” (four items), “stress coping” (four items), “healthy weight” (four items), “safe living environment” (three items), and “vaccinations” (three items), respectively.

**Table 2:** Factor structure of the health literacy behavior and skills scale for preconception care (*n* = 2000)

Item		Factor 1	Factor 2	Factor 3	Factor 4	Factor 5	Factor 6	Commonality
Factor 1 “Appropriate medical examinations”								
1	Are you able to understand instructions provided by doctors or pharmacists on how to use medication prescribed to you?	0.954	−0.016	0.012	−0.053	− 0.042	− 0.061	0.712
2	Do you follow instructions provided by doctors or pharmacists on how to use medication?	0.919	0.007	0.029	−0.062	− 0.032	− 0.146	0.673
3	Are you able to ask a doctor or pharmacist for clarification when his or her explanation is unclear to you?	0.915	−0.047	−0.007	0.027	−0.026	− 0.047	0.696
4	When a doctor has presented multiple treatment methods to choose from, are you able to assess each of their advantages and disadvantages?	0.644	0.044	−0.053	0.081	0.024	0.123	0.643
5	When you have misgivings about a diagnosis or treatment presented to you, are you able to speak to the healthcare professional(s) to communicate your wish to get a second opinion?	0.564	0.012	−0.018	0.047	0.060	0.143	0.570
6	Do you know the diseases that members of your family have suffered from in the past or currently?	0.555	0.046	−0.004	0.061	0.020	0.120	0.485
7	Do you read the explanatory leaflets attached to medication that you purchased on your own?	0.393	0.028	0.033	0.024	0.181	0.072	0.367
Factor 2 “Appropriate diet”								
8	Do you make it a habit to take nutritionally well-balanced meals?	−0.015	0.926	0.002	−0.021	− 0.042	− 0.019	0.630
9	Do you make it a habit to consume plenty of fruits or vegetables?	−0.007	0.857	−0.017	− 0.048	− 0.018	−0.027	0.548
10	Are you able to determine whether a meal is nutritionally well-balanced or not?	0.070	0.738	−0.015	−0.017	0.004	0.002	0.535
11	Do you know the amount of food intake that is best for your own body?	−0.034	0.525	0.068	0.079	0.073	0.048	0.469
Factor 3 “Stress coping”								
12	Do you regularly do things to reduce stress (e.g., getting plenty of rest, exercise)?	−0.033	0.016	0.928	−0.012	0.005	−0.045	0.675
13	Do you have your own ways of dealing with stress?	0.048	−0.034	0.904	−0.068	−0.040	0.017	0.642
14	Do you engage in activities to enrich your inner life and improve your mental state (e.g., meditation, exercise, walking, yoga)?	−0.135	0.036	0.584	0.163	0.099	0.035	0.499
15	Is there someone to help you when you are facing difficulties or you are in trouble?	0.236	0.022	0.527	−0.046	−0.044	0.057	0.451
Factor 4 “Healthy weight”								
16	Do you know your Body Mass Index value?	−0.053	−0.039	− 0.058	0.873	− 0.056	0.073	0.521
17	Do you measure your weight on a regular basis?	0.069	−0.063	−0.002	0.842	−0.072	− 0.054	0.523
18	Do you consistently make efforts to maintain your ideal weight?	−0.002	0.058	0.026	0.733	0.047	− 0.061	0.551
19	Are you able to find information on ways to prevent or deal with lifestyle diseases on your own?	0.130	0.090	0.077	0.495	0.098	0.025	0.604
Factor 5 “Safe living environment”								
20	Do you pay attention to additives when buying food products?	−0.045	0.033	−0.045	− 0.022	0.960	− 0.035	0.657
21	When buying food products, do you pay attention to their places of production?	0.065	−0.063	0.015	−0.048	0.922	−0.084	0.623
22	Do you obtain daily information on air pollution (e.g., PM 2.5) and avoid going out to places with high levels of pollution concentration?	−0.041	0.027	0.043	−0.003	0.502	0.157	0.389
Factor 6 “Vaccinations”								
23	Have you researched information regarding the side effects of a vaccination on your own?	0.013	−0.037	− 0.032	− 0.030	0.067	0.874	0.584
24	Do you use your maternity record book to record and keep track of vaccines taken?	−0.039	0.007	0.025	−0.043	− 0.079	0.866	0.516
25	Do you get flu shots every year?	−0.010	0.005	0.030	0.087	−0.047	0.343	0.139

Factor extraction: maximum likelihood method

Rotation Method: Promax Method with Kaiser Normalization

The cumulative contribution ratio of the six factors in the above factor analysis was 68.9%. The eigenvalue of Factor 6 was above 1 (1.052), but below 1 (0.810) for Factor 7.

In addition, a shortened version of this scale, with a reduced number of items, was examined (*n* = 2000). We examined a model of the shortened version of the scale with items selected based on a factor loading of ±0.6, preserving the six-factor structure of the original 25-item total scale. After factorial loading and validity were investigated, and the number of items was reduced, a shortened version comprising 17 items relating to people’s behavior and skills was obtained (Table 3).

**Table 3:** Shortened factor structure of the health literacy behavior and skills scale for preconception care (n = 2000)

Item		Factor 1	Factor 2	Factor 3	Factor 4	Factor 5	Factor 6	Commonality
Factor 1 “Appropriate medical examinations”								
1	Are you able to understand instructions provided by doctors or pharmacists on how to use medication prescribed to you?	0.899	−0.002	− 0.003	0.005	− 0.022	0.036	0.691
2	Do you follow instructions provided by doctors or pharmacists on how to use medication?	0.874	0.028	−0.004	0.005	−0.021	− 0.045	0.659
3	Are you able to ask a doctor or pharmacist for clarification when his or her explanation is unclear to you?	0.787	−0.012	0.020	0.002	0.081	0.024	0.640
Factor 2 “Appropriate diet”								
4	Do you make it a habit to take nutritionally well-balanced meals?	− 0.013	0.914	−0.028	0.006	− 0.004	− 0.006	0.621
5	Do you make it a habit to consume plenty of fruits or vegetables?	−0.012	0.814	0.000	−0.008	−0.013	− 0.010	0.546
6	Are you able to determine whether a meal is nutritionally well-balanced or not?	0.064	0.661	0.037	0.013	0.007	0.021	0.492
Factor 3 “Stress coping”								
7	Do you pay attention to additives when buying food products?	−0.041	0.042	0.920	−0.030	− 0.011	− 0.017	0.652
8	When buying food products, do you pay attention to their places of production?	0.071	−0.051	0.898	0.009	−0.031	−0.060	0.619
9	Do you obtain daily information on air pollution (e.g., PM 2.5) and avoid going out to places with high levels of pollution concentration?	−0.035	0.021	0.493	0.038	0.029	0.158	0.376
Factor 4 “Healthy weight”								
10	Do you regularly do things to reduce stress (e.g., getting plenty of rest, exercise)?	−0.003	0.008	−0.017	0.963	−0.032	− 0.037	0.670
11	Do you have your own ways of dealing with stress?	0.081	−0.023	−0.031	0.838	−0.049	0.025	0.621
12	Do you engage in activities to enrich your inner life and improve your mental state (e.g., meditation, exercise, walking, yoga)?	−0.106	0.048	0.098	0.568	0.143	0.032	0.483
Factor 5 “Safe living environment”								
13	Do you measure your weight on a regular basis?	0.065	−0.043	−0.047	−0.005	0.850	−0.045	0.519
14	Do you know your Body Mass Index value?	−0.046	−0.013	− 0.022	−0.035	0.831	0.061	0.506
15	Do you consistently make efforts to maintain your ideal weight?	0.025	0.083	0.075	0.049	0.633	−0.035	0.498
Factor 6 “Vaccinations”								
16	Do you use your maternity record book to record and keep track of vaccines taken?	−0.007	0.010	−0.078	0.015	−0.014	0.882	0.506
17	Have you researched information regarding the side effects of a vaccination on your own?	0.025	−0.014	0.107	−0.019	0.010	0.779	0.562

Factor extraction: maximum likelihood method

Rotation Method: Promax Method with Kaiser Normalization

### Participants’ sex and history of pregnancy and childbirth related differences

**Fig. 1:**
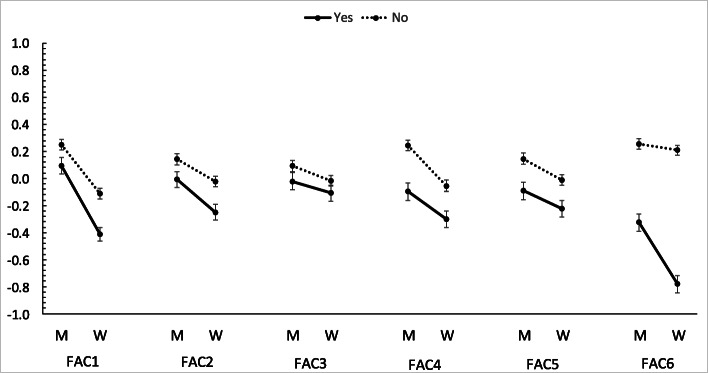
Gender and a history of pregnancy and childbirth related differences in subscale scores M: Men. W: Women. Yes/No: History of pregnancy and childbirth. FAC: Factor. *Higher scores indicate a greater tendency for a lack of health literacy

Figure 1 presents participants’ sex and history of pregnancy and childbirth related differences in the subscale score of behavior and skills scale (participants who or whose partners were pregnant [*n* = 43] were excluded). In total, men scored higher than women and, in terms of history of pregnancy and childbirth, “No” scored higher than “Yes,” indicating a tendency for a lack of health literacy in men and among participants who had no history of pregnancy and childbirth. Regarding the subscale scores on “appropriate medical examinations” (Factor 1), “healthy weight” (Factor 4), “vaccinations” (Factor 6), sex and a history of pregnancy and childbirth related differences were consistently significant in the first and second surveys, with sex and a history of pregnancy and childbirth interacting significantly with “vaccinations” (F (1, 1953) = 16.34, *p* < 0.001; results for Total (*n* = 2000)) (Additional file 6).

### IRT analysis results for knowledge of preconception care items (knowledge scale)

In total, 13 items around knowledge of preconception care, which asked respondents to choose a correct answer, were scaled using the IRT (Table 4) (Additional file 4 shows the knowledge question and answer items). Three other items about whether participants had a regular doctor, received regular health screenings, and had a dental treatment plan were excluded from the analysis, due to considerations around content validity and the low factor loading in the results of the factor analysis. Additionally, after examining only the factor loadings, two items about folic acid intake demonstrated low factor loadings. However, these questions pertained to the respondents’ knowledge and were considered essential to the analysis. Therefore, these questions were retained.

**Table 4:** Analytical results based on item response theory of the knowledge scale for preconception care (total *n* = 2000)

Items		% correct	%correct (Men)	%correct (Women)	Slope	S.E. of slope	Threshold	S.E. of threshold
		Total	Total	Total	Total	Total	Total	Total
1	Which of the following is a mistaken approach to birth control?	0.586	0.521	0.650	0.397	0.040	−0.858	0.091
2	Which of the following is incorrect about birth control pills?	0.737	0.704	0.770	0.425	0.045	−1.879	0.161
3	Which of the following is incorrect about pregnancy?	0.691	0.649	0.732	0.653	0.050	−1.196	0.071
4	What is the minimum amount of folic acid that should be taken to effectively reduce the risk of congenital abnormalities of the brain and spinal cord of the baby?	0.166	0.132	0.200	0.234	0.044	3.904	0.750
5	Regarding folic acid intake by women planning to become pregnant, which of the following is correct?	0.691	0.605	0.776	0.129	0.039	−3.993	1.134
6	Which of the following is effective in preventing sexually transmitted diseases?	0.922	0.906	0.937	0.759	0.077	−2.691	0.178
7	Regarding smoking during pregnancy, which of the following is correct?	0.877	0.837	0.917	1.185	0.089	−1.843	0.075
8	Regarding how drinking by the mother during pregnancy can affect the baby, which of the following is correct?	0.897	0.879	0.915	0.691	0.071	−2.556	0.177
9	In terms of places to receive consultation if you have been physically abused by your partner, which of the following is correct?	0.859	0.828	0.891	1.399	0.111	−1.648	0.063
10	What should you do if a symptom of a disease that worries you persists?	0.788	0.776	0.799	0.694	0.055	−1.695	0.100
11	Which of the following hormones is unrelated to the menstrual cycles of women?	0.414	0.357	0.472	0.333	0.038	0.365	0.108
12	Regarding symptoms related to the menstrual cycles of women, which of the following is incorrect?	0.701	0.620	0.783	1.030	0.074	−1.039	0.049
13	Regarding the menstrual cycles of women, which of the following is incorrect?	0.722	0.636	0.808	1.078	0.077	−1.108	0.050
	Mean	0.696	0.650	0.742	0.693	–	−1.249	–

We examined the item parameters, including the discrimination (slope) and difficulty (threshold) of each item under the assumption of 2PL. The Cardiff Fertility Knowledge Scale score was scaled using IRT (Additional file 5), along with comparing the item parameters (discrimination/difficulty). Our 13-item knowledge scale had a lower mean for the item discrimination parameter than the Cardiff Fertility Knowledge Scale (0.693 vs. 0.897). However, the item difficulty parameter of our 13-item knowledge scale varied from approximately − 4.0 to 3.9, while that for the Cardiff Fertility Knowledge Scale ranged only from − 0.8 to 1.3.

As significant difference was found in the correct response rate for each item between men and women, we analyzed the data by sex. We calculated the IRT ability parameters and found that, for our 13-item knowledge scale, the mean for women was 0 (set as reference; SD = 0.787) compared with − 0.388 (SD 0.867) for men. In all 13 of our items, men scored lower than women (higher scores indicating higher knowledge).

### Scale reliability and validity

#### Internal consistency and measurement error

We obtained relatively high reliabilities for the 25-item behavior and skills scale with the total score (0.935) and for all six factor scales (Factor 1 = 0.898, Factor 2 = 0.854, Factor 3 = 0.852, Factor 4 = 0.852, Factor 5 = 0.823, and Factor 6 = 0.686) even though there were fewer items in the lowest factor. Further, we obtained relatively high reliabilities for the shortened version with the 17-item total score (0.910) and for all six factor scales (Factor 1 = 0.900, Factor 2 = 0.846, Factor 3 = 0.823, Factor 4 = 0.848, Factor 5 = 0.823, and Factor 6 = 0.821). The CCHL scale had a high Cronbach’s α value of 0.936.

Values of Cronbach’s α for the 13-item knowledge scale was 0.661 (standard error of measurement was 1.388), and 0.831 for the Cardiff Fertility Knowledge Scale (standard error of measurement was 1.472). Although the 13-item knowledge scale had a lower reliability score, there was almost no difference in measurement error.

#### Criterion-related validity

Table 5 presents the correlation coefficients between the subscale and the total scores of each scale. In total, six factor scores and total scores of behavior and skills scale (6 factors: “appropriate medical examinations,” “appropriate diet,” “stress coping,” “healthy weight,” “safe living environment,” and “vaccinations”) were significantly correlated with the 13-item knowledge scale and the Cardiff Fertility Knowledge Scale scores, with an exception in the correlation between “vaccinations” and the 13-item knowledge scale scores for women (Table 5). There was almost no correlation between the CCHL (a 5-item health literacy scale) and other scales (subscale and total scores of 25-item behavior and skills scale, 13-item knowledge scale, and Cardiff Fertility Knowledge Scale).

**Table 5:** Correlations between factors and scales for health literacy and preconception care (total *n* = 2000)

	Men										Women									
	Factor 1	Factor 2	Factor 3	Factor 4	Factor 5	Factor 6	Overall	CCHL	13-item	CFKS-J	Factor 1	Factor 2	Factor 3	Factor 4	Factor 5	Factor 6	Overall	CCHL	13-item	CFKS-J
Factor 1	1	.532**	.575**	.575**	.516**	.389**	.761**	0	−.355**	−.375**	1	.435**	.429**	.498**	.368**	.395**	.702**	.081*	−.339**	−.327**
Factor 2	.532**	1	.621**	.548**	.616**	.486**	.808**	.063*	−.141**	−.288**	.435**	1	.525**	.525**	.470**	.375**	.757**	.080*	−.120**	−.259**
Factor 3	.575**	.621**	1	.578**	.558**	.455**	.806**	.053	−.197**	−.281**	.429**	.525**	1	.481**	.456**	.387**	.741**	.063*	−.134**	−.196**
Factor 4	.575**	.548**	.578**	1	.563**	.516**	.801**	.05	−.124**	−.298**	.498**	.525**	.481**	1	.447**	.406**	.766**	.052	−.165**	−.262**
Factor 5	.516**	.616**	.558**	.563**	1	.581**	.814**	.023	−.080*	−.247**	.368**	.470**	.456**	.447**	1	.479**	.732**	.086**	−.074*	−.201**
Factor 6	.389**	.486**	.455**	.516**	.581**	1	.725**	.089**	.088**	−.171**	.395**	.375**	.387**	.406**	.479**	1	.700**	.110**	−.027	−.218**
Overall	.761**	.808**	.806**	.801**	.814**	.725**	1	.059	−.172**	−.352**	.702**	.757**	.741**	.766**	.732**	.700**	1	.108**	−.191**	−.332**
CCHL	0	.063*	.053	.05	.023	.089**	.059	1	.108**	.05	.081*	.080*	.063*	.052	.086**	.110**	.108**	1	.082**	−.064*
13-item	−.355**	−.141**	−.197**	−.124**	−.080*	.088**	−.172**	.108**	1	.362**	−.339**	−.120**	−.134**	−.165**	−.074*	−.027	−.191**	.082**	1	.327**
CFKS-J	−.375**	−.288**	−.281**	−.298**	−.247**	−.171**	−.352**	.05	.362**	1	−.327**	−.259**	−.196**	−.262**	−.201**	−.218**	−.332**	−.064*	.327**	1

*p < 0.05、**p < 0.01

Factors 1–6: higher scores indicate a greater tendency for a lack of health literacy

Overall (25-item total score): higher scores indicate a greater tendency for a lack of health literacy

CCHL: Communicative and Critical Health Literacy scale, with higher scores indicating higher health literacy

Our 13-item scale assessing preconception care knowledge, with higher scores indicating higher knowledge

CFKS-J: the Japanese version of the Cardiff Fertility Knowledge Scale, with higher scores indicating higher knowledge

## Discussion

In this study, we developed a 6-factor (“appropriate medical examinations,” “appropriate diet,” “stress coping,” “healthy weight,” “safe living environment,” and “vaccinations”) 25-item behavior and skills scale, as well as a 13-item knowledge scale, to evaluate people’s health literacy in terms of preconception care. The final 25 items were matched in the different populations of the first and second surveys. A short version, consisting of 17 items, was also created from the 25-item behavior and skills scale. The item difficulty parameter of our 13-item knowledge scale showed wider variability than that of a previous knowledge scale [21], meaning that this would allow a more accurate measurement of the extent of participants’ preconception care knowledge, especially for those with extremely low knowledge in this area and consequently, higher needs for health promotion.

The reliability coefficient of the total scores and each factor of the behavior and skills scale was comparatively high, with weak-to-moderate correlation between our behavior and skills scale and knowledge scale. In this study, there was almost no correlation between the CCHL (a 5-item health literacy scale) and our scales, including the 25-item behavior and skills scale and the 13-item knowledge scale, as well as Cardiff Fertility Knowledge Scale. We adopted CCHL for criterion-related validity verification because it was validated in Japanese and contained a relatively small number of items. However, the CCHL may not cover the whole concept of the communicative and critical health literacy [14], and further research with other validated health literacy instruments, such as HLS-EU-Q [13], is required to examine our scale’s validity as a health literacy scale.

Regarding the subscales of the behavior and skills scale, men scored higher than women, and participants with a history of pregnancy and childbirth scored “No” more often than “Yes.” This indicated higher needs for health promotion among men and participants with no history of pregnancy and childbirth. Although this study involved participants from a wide range of age groups including teenagers, not having a pregnancy history does not necessarily mean low health literacy. Additionally, for the knowledge scale, across all 13 items, men scored lower than women. These results are consistent with our hypothesis and suggest that we need to focus on these particular groups in future preconception care programs.

Poor nutrition and obesity among reproductive age women are common problems in both low- and high-income countries, and few interventions related to both diet and lifestyle during the pre-pregnancy period are available [2]. From a preventive medicine perspective, it is important to adequately inform both men and women before pregnancy about the potential impact of lifestyle factors, such as smoking, drinking alcohol, nutrition, infectious diseases, and exposure to pollutants, on their reproductive and future maternal and child health. In addition, more risk assessment and reduction programs need to be provided. The health literacy scale, consisting of both behavior and skills scale and knowledge items, for preconception care developed in this study, will provide information about preconception health to the respondents and support the effective implementation of risk assessment and interventions to promote healthy behavioral changes.

### Limitations and implications

#### Conceptual issues

In this study, we developed a scale for a wide range of age groups (from adolescents to people in their 40s) for both men and women, with the aim of using it in a universal range of environments, such as schools and workplaces. It has been discovered that a population-level approach is important because it is necessary to make long-term efforts to improve people’s appropriate body weight; it is especially ideal to establish appropriate lifestyle habits from adolescence [2]. In addition, although the WHO recommends male involvement in the health promotion dimension of preconception care [1], previous studies on preconception care have found little risk assessment, screening, or other preconception interventions for men [7, 22]. Additionally, our results demonstrate that men score poorer results than women in all subscales of the behavior and skills scale and knowledge scale items, indicating the need for specific preconception health promotion among men.

Conversely, this scale could not include gender-specific reproductive health issues, such as menstrual cycle and self-care. In addition to the universal approach adopted in this study, further research is needed to measure specific risks, such as underlying diseases or detrimental socioeconomic attributes, in both clinical setting and outreach programs [23]. Depending on an individual’s life stage, lifestyle, and level of health literacy, appropriate intervention programs may differ. In future, it will be necessary to identify targeted populations that specifically require preconception care, which will then facilitate the creation of an item pool and development of a behavior and knowledge scale targeting such populations.

#### Methodological issues

This study has the following limitations. First, participants in this study were recruited from registered members of a web-based research company, which could lead to a selection bias. Although the internet usage rate (individual) was over 90% for each age group between 13 and 69 in 2019 in Japan [24], internet surveys involving only registered members of a web-based research company are not guaranteed to be representative of the sample, and we could not know the difference between health literacy of registered members and the rest of the population. Muscat et al. suggested assessing the administration time among a diverse sample, and to use strategies to increase engagement (e.g., use of images and different question formats), as well as to consider use of alternative administration formats (e.g., paper vs online) for the development of health literacy instruments [25]; however, this was not evaluated in this study. Therefore, future research should compare the scale scores among diverse populations, and the scale’s reliability and validity as a health literacy scale compared with standard health literacy measures, such as HLS-EU-Q [13], must also be examined. Furthermore, Muscat et al. suggested involving consumers in the development of health literacy instruments and examining whether consumers consider the instrument as an acceptable and meaningful assessment of their health literacy skills [25]; future research is also needed to capture the opinions of respondents. Finally, to use this scale as a screening tool for preconception health problems, a longitudinal design is needed to determine the cut-off point in clinical settings.

## Conclusions

In this study, we developed a 6-factor 25-item behavior and skills scale, as well as a 13-item knowledge scale, to evaluate people’s health literacy for preconception care. A shortened version with 17 items was also prepared using the 25 items of the behavior and skills scale. The use of this scale will allow both researchers and health professionals to understand the current state of health literacy around preconception care in the general populace. In addition, this preconception care health literacy scale, which consists of behavior and knowledge dimensions, will support the effective implementation of risk assessment and intervention programs aimed at promoting adaptive behavioral changes that utilize a population-based approach.

## Supplementary Information

**Additional file 1.** File extension .xlsx. Characteristics of study participants. A table showing the characteristics of this study’s participants for the first and second surveys separately

**Additional file 2 **File extension .xlsx. Factor structure of the health literacy behavior and skills scale for preconception care (First survey *n* = 1000). A table showing the various factors and items of this scale, following factor extraction

**Additional file 3.** File extension .xlsx. Factor structure of the health literacy behavior and skills scale for preconception care (Second survey n = 1000). A table showing the factors and items of this scale, following factor extraction

**Additional file 4.** File extension .xlsx. Knowledge scale items. A table listing the items of the knowledge scale for preconception care developed by this study

**Additional file 5 **File extension .xlsx. Analytical results for the Cardiff Fertility Knowledge Scale based on item reaction theory (total *n* = 2000). A table showing the results of responses to this scale, based on the correct and incorrect responses by male versus female participants

**Additional file 6.** File extension .xlsx. ANOVA results for sex and history of pregnancy and childbirth related differences in health literacy behavior and skills scale for preconception care. A table showing the results of the ANOVA based on these variables

**Additional file 7..** File extension. Docx. COSMIN reporting guideline. A table showing the COSMIN reporting guideline check items

## Data Availability

The datasets used and/or analyzed during the current study are available from the corresponding author on reasonable request.

## Abbreviations

CDC: Centers for Disease Control and Prevention
CCHL: Communicative and Critical Health Literacy
COSMIN: Consensus-based Standards for the Selection of Health Status Measurement Instruments
HLS-EU-Q: European Health Literacy Survey Questionnaire
IRT: Item response theory
SD: Standard deviation
2PL: Two-parameter logistic model
WHO: World Health Organization
