# Supplementary material for: Development of a health literacy scale for preconception care: a study of the reproductive age population in Japan
Source: BMC Public Health. 2021 Nov 10;21:2057. doi: 10.1186/s12889-021-12081-0 (PMC8579655; doi:10.1186/s12889-021-12081-0)
Supplement: Supplementary file 7 — Additional file 7.. File extension. Docx. COSMIN reporting guideline. A table showing the COSMIN reporting guideline check items [file 12889_2021_12081_MOESM7_ESM.docx]

COSMIN reporting guideline

Gagnier JJ, Lai J, Mokkink LB, Terwee CB. COSMIN reporting guideline for studies on measurement properties of patient-reported outcome measures. Qual Life Res. 2021 Aug;30(8):2197-2218. doi: 10.1007/s11136-021-02822-4. Epub 2021 Apr 5. PMID: 33818733.

| General reporting recommendations relevant for all studies on measurement properties | | | |
| --- | --- | --- | --- |
| Item number | Item name | Item description | Location in manuscript where items are reported |
| Report section: title |  |  |  |
| T1 | Patient-reported outcome measure (PROM) | The name of the PROM instrument(s) (and version if relevant) being  studied | Title |
| T2 | Measurement property (MP) | What MPs are being studied or more generally, that MPs are being studied (if there are many properties being investigated, for example) | Title |
| T3 | Study sample | General description of relevant study sample characteristics (e.g., condition of interest, language) and also any intervention or exposure (e.g., treatments) if applicable | Title |
| Report section: abstract |  |  |  |
| A1 | PROM | The name of the PROM instrument(s) (and version if relevant) being studied (i.e., the SF-36 or SF-12; language version) or if it concerns an item bank (e.g., PROMIS instruments). The type of instrument (e.g., a self-reported questionnaire or interview) | Abstract |
| A2 | Measurement property | What MPs are being studied or more generally, that MPs are being studied (if there are many properties being investigated, for example) | Abstract |
| A3 | Design | The type of study being used to test the properties (e.g., test–retest design, longitudinal study, cohort, cross sectional, case series, randomized etc.). Other details of the study design if relevant (intervention/ exposure, description of comparison instruments, outcomes other than PROMs) | Abstract |
| A4 | Sample | Inclusion / exclusion criteria. General description of relevant study sample characteristics (e.g., condition of interest, geographic location, language, other relevant demographic and baseline characteristics) | Abstract |
| A5 | Methods | A brief description of the methods for investigating each MP including statistical analyses | Abstract |
| A6 | Results | The main results for all MPs investigated reporting statistics for each result with measures of precision where appropriate | Abstract |
| A7 | Discussion/Conclusions | A brief description of the results in the context of existing evidence, main strengths and drawbacks and the need for future research on the PROM(s) investigated | Abstract |
| Report section: introduction |  |  |  |
| I1 | Name and describe the PROM of interest | Specify the name, type, language, and version of the PROM being investigated and how it was developed. Describe the construct the PROM aims to measure and its subscales; describe the structure of the PROM (e.g., the number of factors, the number of items, scoring algorithm); describe relevant instructions (like time period), and number or type of response categories. State whether the PROM is based on a reflective or formative model | Background |
| I2 | Target population | Describe the specific target population that the PROM was designed for. The authors need to provide the appropriate and necessary characteristics of this population | Background |
| I3 | Citation for the original development of the PROM | The citation for the original development paper(s) should be provided and other highly relevant citations related to the quality of the specific PROM under investigation | Background |
| I4 | State of knowledge & Rationale | A description of the current scientific knowledge (what is known) regarding the MPs of? the PROM under investigation. The authors should provide a literature review or refer to a recent review of all existing evidence of the specific version (e.g., language, short form) of the PROM and explain why the new study is necessary and important. The rational for the current proposed study should be given | Background |
| I5 | Definitions | Specialized terms should be defined or explained | Background |
| I6 | Objectives and hypotheses | State the specific objective(s) of the research and hypotheses related to the specific PROM under investigation | Background |
| Report section: general methods |  |  |  |
| GM1 | Study design | State the key elements of the study design | Methods |
| GM2 | Participants | State how the participants were chosen; the inclusion and exclusion criteria. (e.g., if a PROM for a specific condition, then the eligibility and selection criteria should reflect this) | Methods |
| GM3 | PROM administration | An explicit description of how and when the PROM(s) were administered (e.g., in what setting) including data collection devices/system used (e.g., paper-based, electronic administration / ePRO) should be provided | Methods |
| GM4 | Data collection procedures | Provide information about other data collection, exposure methods (e.g., allocation to interventions) and time points / follow-up points | NA |
| GM5 | Power/sample size calculation | Provide a power calculation for all MP analyses. Alternatively, if a rule of thumb is used, state it and the source/citation | NA |
| GM6 | Statistical analyses | Statistical analyses and tests corresponding to all hypotheses or objectives for all MPs should be reported. Where appropriate, a cutoff for statistical significance should be reported (e.g., p-value less than 0.05). A description of all statistics to be used to estimate the magnitude and direction of effect should also be reported, together with measures of variability or precision. Report statistical package used | Methods |
| GM7 | Missing data | State approaches or plan for dealing with missing data | NA |
| GM8 | Post hoc analysis | The report should specify analyses that used data after the data collection period concluded (i.e., if the analyses were post hoc; secondary data analyses) and describe the rationale for any post hoc analyses | Methods |
| Report section: general results |  |  |  |
| GR1 | Missing data | The amount and reasons for missing data should be explained for all analyses for all PROMs (or other outcome measurement instruments) and relevant groups | NA |
| GR2 | Participant/patient Characteristics | The study patients’ characteristics should be described, including baseline PROM scores | Results |
| GR3 | Sample size | If one study contained analyses using different sample sizes, the authors should report the sample size for each analysis | Results |
| Report section: discussion |  |  |  |
| D1 | MP evidence | Per measurement property the authors should compare the result to the criteria for good measurement properties (e.g., COSMIN criteria) [25], and determine if the specific MP is sufficient or not. Note: This information may also appear in the results section in greater detail in a table for example | Discussion |
| D2 | Practical relevance | The authors need to discuss the practical relevance of the findings | Discussion |
| D3 | Strengths and limitations | Strengths and limitations of the study should be discussed. For example, discuss if there were any significant potential biases in the study that could have impacted the results | Discussion |
| D4 | Generalizability | Generalizability issues related to the PROM results should be discussed. For example, discuss if the results could be generalized to other populations given the sample studied | Discussion |
| D5 | Instrument changes | Discuss the need for modifications to the existing PROM or new PROM development. If you conclude that one of the measurement properties is insufficient, you could suggest some modification, or if it is really poor, you could suggest stopping use of the PROM (in the specific population or in general) | Discussion |
| D6 | Future research | Report specifically the type of research needed to answer new questions arising out of these findings for the particular MP and PROM investigated | Discussion |
| Report section: conclusions |  |  |  |
| C1 | Conclusions | State the overall conclusions for each MP and of the use PROM investigated | Conclusion |
| Report section: other information |  |  |  |
| O1 | Conflict of interest | State any relevant conflict of interest related to the PROM under investigation (e.g., an author being the PROM developer, funding body etc.) | Competing interests |
| Specific reporting recommendations for studies on content validity | | | |
| CV1 | Relevance | Report if and how patients and/or professionals were asked whether each item is relevant for their experience with the condition | Methods |
| CV2 | Comprehensiveness | Report if and how patients and/or professionals were asked whether all key concepts are included | Methods |
| CV3 | Comprehensibility | Report if and how the comprehensibility of the PROM instructions, items, response options, and recall period was assessed | Methods/ Limitation |
| CV4 | Relevance results | Report if all items were considered relevant for the construct, population, and context of use of interest by patients and/or professionals | Methods |
| CV5 | Response options and recall period | Report whether the response options and recall period were considered appropriate by patients and/or professionals | Methods/ Limitation |
| CV6 | Comprehensiveness results | Report whether patients and/or professionals considered all key concepts to be included in the PROM | Methods |
| CV7 | Comprehensibility results | Report whether patients understood the PROM instructions, items, and response options as intended | Limitation |
| Specific reporting recommendations for studies on structural validity | | | |
| SV1 | Factor analyses: classical test theory (CTT) PROMs | Report details of the methods and results for any exploratory or confirmatory factor analyses. State the rational for any explorative factor analyses (e.g., no clear a priori hypotheses). For CFA, describe and justify the factor structure of tested models. Methods and results for checking of the assumptions should be described, the method of estimation, goodness-of-fit statistics and cutoff points for good model fit, including factor loadings of best-fitting model | Methods/ Results |
| SV2 | Item Response theory (IRT) analyses | Type of IRT/Rasch model should be reported. Also report the method of estimation, methods and results for checking of the assumptions (unidimensionality (see factor analysis), local dependency (e.g., residual correlations), monotonicity; (e.g., Mokken scaling), goodness-of-fit statistics, and cutoff points for goodness of item/model fit, and all item parameters | Methods/ Results |
| Specific reporting recommendations for studies on internal consistency | | | |
| IC1 | Unit of measurement | Report internal consistency methods and results for each unidimensional scale or subscale. Report all evidence or assumptions associated with unidimensionality | Methods/ Results |
| IC2 | Continuous scores | Report Cronbach’s alpha or omega statistics. Report other statistics calculated for internal consistency of continuous scores | Methods/ Results |
| IC3 | Dichotomous scores | Report Cronbach’s alpha or Kuder–Richardson coefficient. Report other statistics calculated for internal consistency of dichotomous scores | Methods/ Results |
| Specific reporting recommendations for studies on cross-cultural validity measurement invariance | | | |
| CCV1 | Comparator group(s) | Report characteristics of (sub)groups being compared. Include sample sizes in each group | Methods/ Results |
| CCV2 | Factor analyses: classical test theory (CTT) PROMs | Report details of the methods and results for multiple-group confirmatory factor analyses, logistic regression analyses, or other analyses performed. Describe and justify the series of tested models, including constraints of factor loadings, intercepts and variances in CFA. Methods and results for checking of the assumptions should be described. Criteria to define invariance. Describe the method of estimation, goodness-of-fit statistics and criteria used to flag items for measurement invariance | Methods/ Results |
| CCV3 | Item response theory (IRT) analyses | Type of IRT/Rasch model should be reported. Also report the methods and results for checking of the assumptions (unidimensionality (see factor analysis), local dependency (e.g., residual correlations), monotonicity; (e.g., Mokken scaling). Describe statistical packages, method of estimation, criteria used to flag items for DIF, and methods and results of all model comparisons | Methods/ Results |
| Specific reporting recommendations for studies on reliability | | | |
| R1 | PROM administrations | Report the total number of measurements made and if the measurements were applied to the same samples using the same PROM. The process of administrating the measurements to the patients should be described, including who administered it (i.e., did the patient complete it or was there a proxy), when, how and any time intervals between administrations should be reported. This should include: time interval between repeated measurements (e.g., was the patient stable or not), the test type (e.g., a self‐administered questionnaire, an interview-based PROM), the setting in which the instrument was administered (e.g., at the hospital, or at home), and the instructions given for completing it. If relevant, other instruments or measurements accompanying the repeated PROM measurement. Also, if relevant, the independence (whether the PROM was completed without knowledge of the previous scores) of the administrations | Methods/ Results |
| R2 | Statistical analyses | All statistical analyses and results specific to the reliability assessment(s) should be described and their use justified (e.g., the intraclass correlation coefficient (ICC) model or type of Kappa coefficient used). Also, describe the variance components, and the weighting scheme used for ordinal scores (e.g., linear or quadratic weights) | Methods/ Results |
| R3 | Methods to improve reliability | Report any methods used to improve reliability such as restriction of the sample, training of researchers and standardization of methods, and averaging of repeated measurements | Methods/ Results |
| Specific reporting recommendations for studies on measurement error | | | |
| ME1 | PROM administrations | Report the total number of measurements made and if the measurements were applied to the same samples using the same PROM. The process of administrating the measurements to the patients should be described, including who administered it (i.e., did the patient complete it or was there a proxy), when, how and any time intervals between administrations should be reported. This should include: time interval between repeated measurements (e.g., was the patient stable or not), the test type (e.g., a self‐administered questionnaire, an interview-based PROM), the setting in which the instrument was administered (e.g., at the hospital, or at home), and the instructions given for completing it. If relevant, other instruments or measurements accompanying the repeated PROM measurement. Also, if relevant, the independence (whether the PROM was completed without knowledge of the previous completion) of the administrations | Methods/ Results |
| ME2 | Statistical analyses | All statistical analyses and results specific to measurement error assessment(s) should be described and their use justified. Specifically, for continuous scores report the Standard Error of Measurement (SEM; Specify the exact model used to calculate the SEM (i.e., SEM consistency or SEM agreement)), Smallest Detectable Change (SDC; specify formula used, included the model of the SEM when based on the SEM) or Limits of Agreement (LoA). For dichotomous/nominal/ordinal scores report marginals (raw data) and the percentage specific (e.g., positive and negative) agreement | Methods/ Results |
| Specific reporting recommendations for studies on criterion validity | | | |
| CriV1 | Criterion | Report the details of the criterion used and why it was used. Justification of the gold standard must be reported. Also, describe (if applicable) how and why the criterion was dichotomized or classified. Also, how and when the criterion was administered (e.g., if independent from the PROM) | Methods/ Results |
| CriV2 | Continuous scores | Report correlations (when criterion has continuous scores) or the area under the receiver operating characteristic (ROC) curve (when criterion is dichotomous) | Methods/ Results |
| CriV3 | Categorical scores | Described how (and why) the PROM was dichotomized or made into multiple categories. Report sensitivity and specificity statistics | Methods/ Results |
| Specific reporting recommendations for studies on hypotheses testing for construct validity | | | |
| ConV1 | Comparator instrument(s) | The comparator instruments should be appropriately described in terms of the construct(s) they intend to measure. Report the measurement properties of the comparator instruments and related citations or data | Methods/ Results |
| ConV2 | Comparator Group(s) | Report characteristics of groups being compared. Include sample sizes in each group | Methods/ Results |
| ConV3 | Hypotheses | Report all hypotheses including the direction and magnitude of the expected correlations between the PROM of interest and another measurement instrument, or the direction and magnitude of differences in scores of the PROM between groups | Methods/ Results |
| ConV4 | Statistical analyses | Report all statistical methods and results used to test each hypothesis | Methods/ Results |
| ConV5 | Results | Report which specific results are in accordance with its hypothesis | Results/ Discussion |
| Specific reporting recommendations for studies on responsiveness | | | |
| Resp1 | Comparison instrument(s) | The comparator instruments should be appropriately described in terms of the construct(s) they intend to measure. Report the measurement properties of the comparator instruments and related citations or data | NA |
| Resp2 | Comparator group(s) | Report characteristics of groups being compared. Include sample sizes in each group | NA |
| Resp3 | Hypotheses | Report all hypotheses including the direction and magnitude of the expected correlations between changes in the PROM of interest and change in another measurement instrument, or the direction and magnitude of differences in change scores of the PROM between groups | NA |
| Resp4 | Measurement procedures | Report if measurements were applied to the same sample using the same instruments. Describe the measurement procedures, including time intervals between different measurement instruments | NA |
| Resp5 | Interim period | The interim period between time points should be described | NA |
| Resp6 | Intervention/exposure | Describe the intervention given or exposure in the interim period if relevant | NA |
| Resp7 | Patients changed | Report the proportion of patients that improved or deteriorated (and the details of any anchor used) on the construct measured on all PROMs. Report any changes in scores of the PROM in the target population for the research application relative to the predefined hypotheses | NA |
| Resp8 | Statistical analyses | Report all statistical methods and results used to test each hypothesis | NA |
| Resp9 | Results | Report which specific results are in accordance with its hypothesis | NA |
